# Abnormal Dynamics of Functional Connectivity Density Associated With Chronic Neck Pain

**DOI:** 10.3389/fnmol.2022.880228

**Published:** 2022-06-29

**Authors:** Xixiu Ni, Jiabao Zhang, Mingsheng Sun, Linjia Wang, Tao Xu, Qian Zeng, Xiao Wang, Ziwen Wang, Huaqiang Liao, Yimei Hu, Qing Gao, Ling Zhao

**Affiliations:** ^1^Acupuncture and Tuina School, Chengdu University of Traditional Chinese Medicine, Chengdu, China; ^2^School of Mathematical Sciences, University of Electronic Science and Technology of China, Chengdu, China; ^3^Department of Imaging, Hospital of Chengdu University of Traditional Chinese Medicine, Chengdu, China; ^4^Department of Orthopedics, Hospital of Chengdu University of Traditional Chinese Medicine, Chengdu, China

**Keywords:** chronic neck pain, dynamic, functional connectivity density, resting-state functional magnetic resonance imaging, negative emotion

## Abstract

**Background**: Chronic neck pain (CNP) is highly prevalent and complicated, associated with limited movement, and accompanied by shoulder pain and other clinical manifestations such as dizziness, anxiety, and insomnia. Brain structural and functional abnormalities often occur in patients with CNP. However, knowledge of the brain’s functional organization and temporal dynamics in CNP patients is limited. Dynamic functional connectivity density (dFCD) can reflect the ability of brain areas or voxels to integrate information, and could become neuroimaging markers for objectively reflecting pain to a certain extent. Therefore, this study compared the dFCD between CNP patients and healthy controls (HCs) and investigated potential associations of the abnormal density variability in dynamic functional connectivity with pain characteristics in CNP patients.

**Methods**: Resting functional magnetic resonance imaging was performed for 89 CNP patients and 57 HCs. After preprocessing resting-state fMRI images by the Data Processing and Analysis of Brain Imaging toolbox, the sliding window method was applied to investigate dFCD changes in CNP patients and HCs using the DynamicBC toolbox. Then we quantified dFCD variability using their standard deviation. Based on the pain-associated factors collected from the case report form of CNP patients, the mean dFCD variability values of each dFCD from region of interest were extracted to calculate Pearson’s correlation coefficient to study the potential correlation between dFCD abnormal variability and pain.

**Results**: Compared with HCs, the dFCD values of the anterior cingulate cortex, occipital lobe, temporal lobe, and cerebellum were statistically different in patients with CNP. Subsequent correlation analysis showed that the variable dFCD in the related brain region was correlative with the course of the disease and clinical symptoms, such as pain and depression, in patients with CNP.

**Conclusion**: Dynamic functional alterations were observed in the brain regions of CNP patients, and the dFCD of these brain regions could become neuroimaging markers for objectively reflecting pain to a certain extent. This suggests that chronic pain may cause changes in pain processing and emotional feedback and highlights the link between dynamic neural communication in brain regions and disease conditions, deepening our understanding of chronic pain diseases, and guiding clinical practice.

## Introduction

Chronic neck pain (CNP), one of the most common chronic pain conditions, is a type of extensive pain characterized as discomfort and stiffness of the neck soft tissue for more than 3 months but no abnormality is observed during imaging or other related examination. It is often associated with limited movement and is accompanied by shoulder pain and other clinical manifestations such as dizziness, anxiety, and insomnia (Borghouts et al., 1998). The prevalence of neck pain in the general adult population worldwide ranges from 30% to 50% (Hogg-Johnson et al., 2009), in particular, 65% of individuals experience neck pain-related diseases in China (Marchesini et al., 2021), and is the third most common condition causing chronic pain in the United States and the fourth leading cause of disability worldwide (Murray et al., 2013; Hoy et al., 2014). Clinical evidence indicates that patients with chronic pain may develop cumulative brain damage because of pain-related process recurrence (Zhao et al., 2013) accompanied by dysfunction in sensory, cognition, memory, and emotional processing (Denkinger et al., 2014). Patients with neck pain often experience serious sleep disturbances (Craig Chloe et al., 2021), negative emotions, such as anxiety and depression (Elbinoune et al., 2016), and psychological reactions caused by repeated attacks and prolonged neck pain (Uthaikhup et al., 2015). Emotional disorders, primarily depression, gradually develop and affect the patients’ confidence and their treatment efficacy (Phan et al., 2017).

Many complex related factors cause neck pain, including general demographic factors (age, gender, race); lifestyle, social and psychosocial factors. CNP is the result of the comprehensive actions of these factors (Lopez-Lopez et al., 2015). The pathogenesis of CNP is complicated and unclear, and the associated pain can be aggravated as it progresses (Visser and van Dieën, 2006). Previous studies have reported the multifactorial pathogenic characteristics of CNP. Some studies have suggested that the main mechanisms of neck pain include cervical muscle strain, resulting in cervical spine biomechanical imbalances, cervical disc degeneration, and cervical spine curvature changes (Yang et al., 2016).

Resting-state functional connectivity (FC), which is measured by functional magnetic resonance imaging (fMRI), can be used to investigate the integration of the functional brain network at rest. Resting-state FC has been widely used during neuroimaging studies to examine cerebral functional alterations in various types of pain such as chronic back (Baliki et al., 2011) and low back pains (Ng et al., 2018), migraine (Yuan et al., 2013), and neck pain (Woodworth et al., 2019). Patients with neck pain experience superior frontal gyrus, anterior cingulate gyrus, precuneus cortical thinning, and decreased putamen volume. These changes are associated with neurological severity, pain severity, cortical thickness, and subcortical volume changes (Woodworth et al., 2019). Two fMRI studies using a resting-state regional homogeneity (ReHo) analysis showed significantly different brain activity in specific brain areas such as the middle frontal gyrus, left insula, and superior frontal gyrus in CNP patients compared with control groups (Yu et al., 2017; Chen et al., 2018). It has been reported that CNP is associated with the right temporo-parietal junction and sensorimotor area dysfunction (Chen et al., 2018). These results suggest that patients with CNP may have somatosensory, cognitive, and motor dysfunction. However, the functional brain networks involved in CNP remain unknown.

Static methods, such as ReHo and FC, have been used to analyze CNP and assume that the brain remains stable over time. To some extent, they lose the timeliness characteristic, which may be an inadequate representation of the human brain plasticity owing to the human brain’s time-dependent and dynamic nature (Li et al., 2019). It is likely that brain signals fluctuate over time during CNP. Human brain connectivity is dynamic and associated with ongoing rhythmic activity rather than stationary activity over time (Calhoun et al., 2014). An emerging area of study is dynamic functional connectivity (dFC), which can be investigated by measuring the variability in strength. Compared with the previous static methods, brain dynamics would reflect the aspects of neural system functional capacity, so dynamic functional connectivity density (dFCD) can reflect the ability of brain areas or voxels to integrate information. A previous study indicated that changes in FC may be related to changes in the mental state (Hutchison et al., 2013). Another study demonstrated that dFC analysis can reveal dynamic brain processes underlying facilitated central pain processing in fibromyalgia (Cheng et al., 2021). Functional brain activity rapidly and dynamically reorganizes over time, and CNP also is characterized by pain time accumulation. Zou suggested that functional features of the chronic migraine brain may fluctuate over time instead of remaining static (Zou et al., 2021). A full understanding of CNP thus requires a dynamic measure that evolves. The destruction of this intrinsic FC through the pathological process of chronic pain may be an important mechanism of brain dysfunction in CNP patients. Therefore, the study of functional dynamics may provide a new perspective regarding abnormal brain communication in CNP. Recently, neuroimaging researchers have shown an interest in the temporal fluctuations in FC. Graph theory analysis can provide a powerful framework to characterize the functional connections within the whole brain network (Bullmore and Sporns, 2009) and functional connectivity density (FCD) mapping is a novel voxel-mode graph theory method used to identify centers in the human brain (Tomasi and Volkow, 2010). Several studies have investigated differences in dFC between healthy controls (HCs) and patients with neuropsychiatric or pain disorders using this approach (Gao et al., 2016; Díez-Cirarda et al., 2017) and showed differences in dFC between patients and HCs. Therefore, measuring the dFCD is a feasible method of evaluating functional changes related to CNP.

We noted that previously published dFCD studies often used psychiatric cohorts, but chronic pain had not received much attention. Our work was different because we studied and compared the dFCD of CNP patients and HCs. Based on previous findings (Xu et al., 2021), compared with HC, CNP patients have a significantly lower volume of pain-related brain regions such as the left anterior cingulate gyrus and right superior gyrus. Therefore, we hypothesized that: (1) CNP patients would exhibit specific dFCD differences in regions associated with pain, and; (2) abnormal dFCD in CNP patients is related to the degrees of pain, neck dysfunction, and emotion. The application of this new dynamic analysis method may better reflect the ability of brain areas or voxels to integrate information and enhance the understanding of central pathological injury caused by CNP by providing multiple perspectives. Similarly, this analysis method can also provide neuroimaging markers for objectively reflecting pain to a certain extent, deepen the understanding of chronic pain diseases, and guide clinical practice. Thus, this study aimed to: (1) combine the FCD method and sliding windows correlation analysis to compare the dFCD of CNP patients and HCs and; (2) investigate the potential associations of abnormal dFCD variability with pain characteristics of CNP patients.

## Materials and Methods

### Ethics Statement

The present research conformed to the principles established in the Declaration of Helsinki and was approved by the Sichuan Regional Ethics Review Committee on Traditional Chinese Medicine(ethical approval number: 2018KL-056). Prior to the study, each participant was informed of the experiment process and related matters and signed an informed consent form. Our research has already been registered on http://www.chictr.org.cn (registration number: ChiCTR1800017718).

### Participants

Fifty-seven HCs were recruited using advertising and 89 CNP patients were recruited from the acupuncture and orthopedic clinics of the Hospital of Chengdu University of Traditional Chinese Medicine.

The criteria for CNP that we used were those compiled by the Orthopedic Section of the American Physical Therapy Association (Blanpied et al., 2017). They included the following: (1) neck pain and discomfort or limited cervical motion as the main symptoms; (2) age 18–75 years; (3) right-hand dominance; (4) had a pain score of >3 cm on a visual analog scale (VAS) for five of seven pretreatment days (range, 0–10 cm); (5) disease course more than 3 months; (6) signed the informed consent. The exclusion criteria were as follows: (1) macroscopic T2-visible brain lesions on MRI scans; (2) complications associated with other serious organic diseases, including malignant tumors, tuberculosis, fracture, and osteomyelitis; (3) complications associated with serious primary diseases, including cardiovascular, cerebrovascular, liver, kidney, and hematopoietic systems; (4) mental disorders and mental disorders that could not be matched with the questionnaire(the score of Self-Rating Anxiety Scale (SAS)or Self-Rating Depression Scale (SDS) reaches greater than 72); (5) bleeding tendencies, allergies, and skin diseases; pregnancy, lactation, and fertility issues within the past 6 months; (6) contraindications such as metals in the body; (7) participation in other simultaneous clinical trials.

The VAS and the short form of the McGill pain questionnaire were used to evaluate the degree of neck pain. The neck disability index (NDI) and the short form 12 (SF-12) questionnaire were used to evaluate the impact of pain intensity and neck pain on daily life. The SDS and SAS were used to assess the emotional status. CNP patients and HCs were matched for age. All participants were provided with information about the research procedure and written informed consent before the experiment.

### MRI Data Acquisition

Using a GE 750 3.0-T MRI system with an eight-channel, phased-array head coil (General Electric, Milwaukee, WI, USA), all participants underwent MRI scanning at the Hospital of Chengdu University of Traditional Chinese Medicine. Participants were asked to rest for 15 min before undergoing scanning. Before entering the MR examination room, the participants removed all metal objects (such as mobile phone, keys, jewelry, glasses, and dentures) and wore elastic foam earplugs or cotton balls to reduce noise during scanning. In the examination room, participants were placed in the supine position, and a coil and sponge pad were fixed to their heads. The participants were asked to close their eyes, relax their bodies, avoid sleeping, and avoid head movement as much as possible. After the structure image was obtained in the three conventional planes, axial scanning of the structure image was performed using the T1-weighted gradient-echo sequence.

The scanning parameters for structure image were as follows: repetition time (TR)/echo time = 2,530 ms/3.4 ms; field of view = 240 mm × 240 mm; matrix size = 512 × 512; turning angle = 12°; layer thickness = 1 mm. The gradient echo sequence of the single-shot planar echo was used for functional imaging. The scanning parameters for function image were as follows: TR/echo time = 2,000 ms/30 ms; matrix = 64 × 64; field of view = 240 mm × 240 mm; turning angle = 90°; slice thickness = 5 mm; continuous scanning without septum; voxel size = 3.75 mm × 3.75 mm × 5 mm. For each participant, 210 volumes were acquired, resulting in a total scan time of 420 s. Scanning was performed for the whole brain, cerebellum, and brainstem.

### Preprocessing of Resting-State fMRI Data

The original DICOM format images were transmitted to the workstation, and the integrity of the original data was checked to ensure that all sequence data of the participants included in the statistical study were complete. The quality of the original data was checked by MRIcron software v1.0.20190902^1^. After eliminating obvious image quality problems, the sufficient image was imported for DICOM-to-NIFTI conversion (dcm2nii), and the image in the DICOM format was transformed into the NII format for statistical analyses. Resting-state fMRI images were preprocessed using the Data Processing and Analysis of Brain Imaging toolbox DPABI, v4.3^2^ as follows: the first 10 volumes were discarded to stabilize the scanner’s signal and enable participants to adapt to the environment; slice timing was corrected for the remaining 200 fMRI images; head motion correction was performed (participants were excluded if their maximal head motion exceeded 3 mm of displacement or 3° of rotation); spatial normalization to the standard Montreal Neurological Institute space and resampling to a resolution of 3 × 3 × 3 mm^3^ were performed; regression of nuisance covariates, including the Friston-24 motion parameters, white matter signals, cerebrospinal fluid signals, and global signal, was performed; spatial smoothing was performed using a Gaussian kernel with full-width at half-maximum of 8 mm; detrending was performed; and temporal bandpass filtering with a frequency band of approximately 0.01–0.08 Hz was performed. No participants exhibited head motions exceeding 3 mm of translation or 3°of rotation in any direction in both groups.

### Analysis of dFCD

The sliding window method was applied to evaluate the dFCD for each participant using the DynamicBC toolbox^3^.

Firstly, Frison used functional connection in the functional imaging field (Friston et al., 1993). FC is the correlation of low-frequency BOLD signals between voxels. The region of interest (ROI) is usually selected as the seed region, and the Pearson correlation coefficient between the average time series of the seed region and the time series of other voxels or brain regions is calculated:


$$r=\frac{\sum_{i=1}^{n}\left[x(i)-\bar{x}\right]\left[y(i)-\bar{y}\right]}{\sqrt{\sum_{i=1}^{n}{\left[x(i)-\bar{x}\right]}^{2}\sum_{i=1}^{n}{\left[y(i)-\bar{y}\right]}^{2}}}$$

Where $\bar{x}$ and $\bar{y}$ are the mean values of time series x(i) and y(i), respectively.

In 2010, Tomasi and Volkow (2010) proposed FCD, a voxel-based data-driven method, which more comprehensively and objectively reflects the average functional connection strength between each voxel and other voxels, and has become a measure of the importance of network nodes. The calculation steps are as follows: (1) Calculate a functional connection matrix by the Pearson correlation method, and then set the threshold. (2) If the value exceeds this threshold, it is considered that there is a connection between the two brain regions. (3) Count the connected brain regions in each brain region as the brain region’s FCD.

dFCD is a brain functional connectivity’s new indicator derived from FC, dFC, and FCD. It uses windows to dynamically intercept signals and calculates the data intercepted by each window. The calculation steps are as follows: (1) Previous studies proposed that the window length is an open, but essential, parameter in sliding window-based resting-state dynamics computation (Li et al., 2018, 2019). To avoid the introduction of spurious fluctuations, the minimum window length should be larger than 1 per f_min_, where f_min_ is the minimum frequency of the correlating time courses (Leonardi and Van De Ville, 2015). A window length of 50 TR was considered the optimal parameter for capturing a rapidly shifting dynamic relationship and obtaining reliable estimates of the correlations between regions (Apkarian et al., 2004; Li et al., 2018). Hence, we selected 50 TR (100 s) as the sliding window length and 1 TR (2 s) as the step size to calculate the dFCD of each participant. (2) Each participant’s time series was divided into 151 windows, and the FCD map was computed within each window, thereby generating a set of FCD maps for each participant. (3) Subsequently, we measured the variance of these maps and used the standard deviation to evaluate the temporal variability of dFCD (dFCD variability). (4) Finally, for each participant, the dFCD variability of each voxel was further transformed into z-scores by subtracting the mean and dividing by the standard deviation of global values.

### Statistical Analysis

SPSS software (v23.0; Chicago, IL, USA) was used for demographic analyses. The independent sample *t*-test and Chi-square test were used to compare the demographic characteristics of the two groups. When *p* < 0.05, the difference was considered statistically significant.

The dFCD variability value was averaged at each voxel across participants within the CNP and HC groups to obtain the dFCD variability distributions of both groups. A two-sample *t*-test was performed to assess the group differences in dFCD variability between the CNP and HC groups, with age used as covariates. Multiple comparisons were corrected using the Gaussian random field method (*p* < 0.05).

### Correlation Analysis

To further investigate the potential associations of abnormal dFCD variability with pain characteristics of patients with neck pain, the mean dFCD variability values of each dFCD ROI were extracted to calculate Pearson’s correlation coefficient using pain-associated factors of neck pain patients, including scores of VAS, McGill, NDI, SF-12, SDS, and SAS. A statistically significant threshold of *p* < 0.05 was set for all correlation analyses.

## Results

### Demographic and Clinical Characteristics of Participants

The demographic characteristics of all participants are summarized in Table 1. There was a significant difference between CNP patients and HCs in terms of age (*p* < 0.05) but no significant difference in terms of gender, height, weight, etc. (*p* > 0.05). Meanwhile, there was a significant difference between the two groups for the score of SF-12, SAS, and SDS (*p* > 0.05; Table 1).

**Table 1:** Demographic characteristics of subjects.

Variable	CNPs (*n* = 89)	HCs (*n* = 57)	*p* value
Age (years)	49 (32.5,56)	25 (25,41.5)	0.000^a^
Sex (M/F)	23/66	10/47	0.242^b^
Education background (years)	13 (9.5,16)	12 (8,16)	0.167^a^
Height (cm)	159 (156,166)	166 (160,170)	0.491^a^
Weight (kg)	56 (50.25,63.50)	56 (50,63)	0.963^a^
BMI	22.03 (20.08,24.22)	21.26 (19.78,23.83)	0.616^a^
Time spent on working at desk per day (hours)	6 (2,8)	4 (2,6)	0.088^a^
Time spent on digital products per day (hours)	3 (2,5)	3.5 (2,5)	0.709^a^
Duration (months)	60 (33,120)	—	—
Pain medication use (yes/no)	4/85	—	—
VAS	5 (4,6.7)	—	—
McGill	18 (14,23)	—	—
NDI	28.94 ± 13.67	—	—
SF-12	31 (28,36)	51 (48,52.25)	0.000^a^
SAS	45 (37,51)	31.25 (27.5,35)	0.000^a^
SDS	42 (33,50)	32.5 (28.75,37.5)	0.000^a^

*All values are median (lower quartile, upper quartile), except that the data of NDI is shown as mean ± standard deviation, and the data of gender and pain medication use are shown as the number of cases; ^a^nonparametric test; ^b^Chi-square test; BMI, Body Mass Index; CNP, chronic neck pain; HC, healthy control; VAS, visual analog scale; NDI, neck disability index; SF-12, short form 12 questionnaire; SAS, self-rating anxiety scale; SDS, self-rating depression scale*.

### dFCD Variability Results

**Figure 1:**
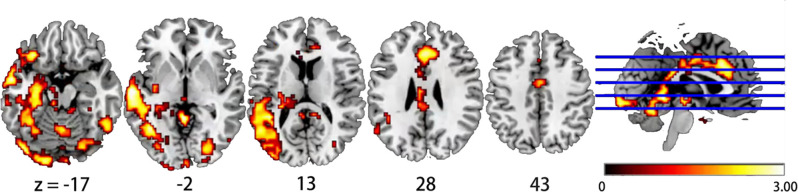
Brain regions showing significant differences in dynamic functional connectivity density (dFCD) variability between the chronic neck pain (CNP) and HC groups. The yellow area represents the difference area, and the deeper the yellow, the greater the difference.

Figure 1 shows the average distribution maps of dFCD variability for each group. The cerebellum, anterior cingulate cortex (ACC), occipital lobe, and temporal lobe had relatively high temporal variances in dFCD values in both groups (Table 2).

**Table 2:** Brain regions showing significant differences in dFCD variability among the two groups.

Brain region	BA	Cluster size	MNI coordinates			*T*-value
			*X*	*Y*	*Z*	
Left anterior cingulate and paracingulate gyrus	31	72	−3	33	21	1.75
Right anterior cingulate and paracingulate gyrus	32	67	6	33	21	2.39
Left medial and paracingulate gyrus	33	91	−3	27	33	2.18
Left fusiform gyrus	55	150	−33	−51	−15	1.77
Left hippocampal gyrus	37	135	−30	−21	−15	2.90
Left inferior occipital gyrus	53	104	−15	−99	−9	2.23
Left middle occipital gyrus	51	163	−36	−69	18	2.46
Left inferior temporal gyrus	89	118	−39	−48	−15	2.07
Right inferior temporal gyrus	90	112	54	−63	−9	1.92
Left middle temporal gyrus	85	627	−60	−15	−3	3.36
Left temporal pole: middle temporal gyrus	87	83	−36	18	−36	2.37
Left superior temporal gyrus	81	218	−60	−15	3	2.15
Right cerebellum superior	100	110	33	−42	−30	2.13
Left cerebellum superior	91	150	−45	−63	−36	1.65
Left cerebellum inferior	93	83	−33	−81	−33	1.86

*Statistical significance level is corrected for multiple comparisons using GRF with *p* < 0.05. The peak coordinate is defined in the MNI space. dFCD, dynamic functional connectivity density; BA, Brodmann area*.

### Correlations

**Figure 2:**
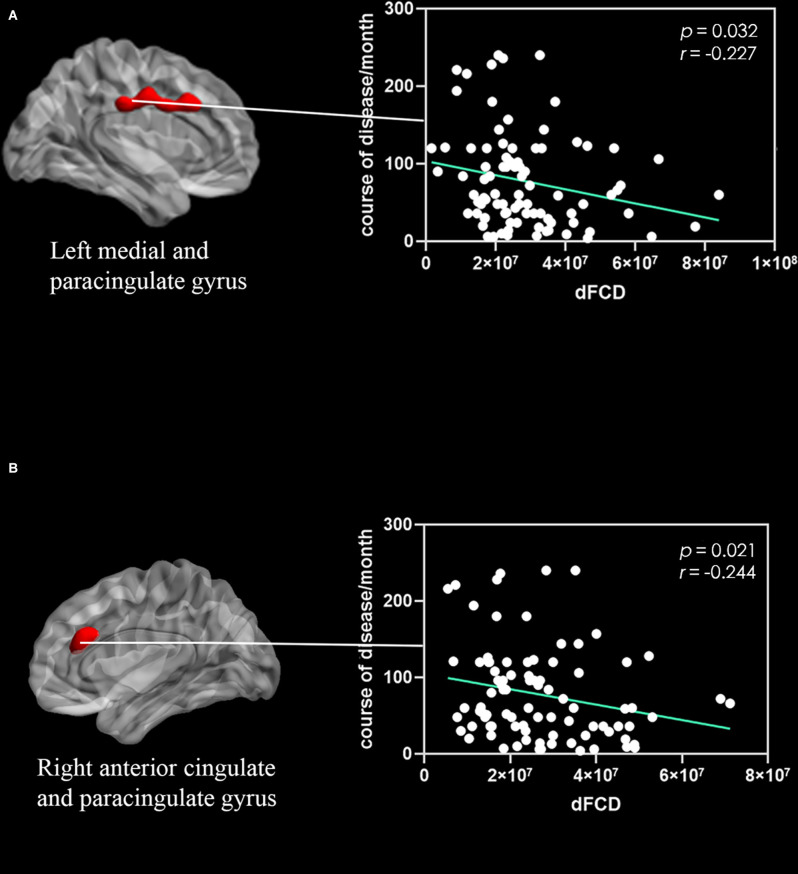
Correlation between dynamic functional connectivity density (dFCD) variability in the left medial and paracingulate gyrus **(A)** and right anterior cingulate and paracingulate gyrus **(B)** with the course of disease in CNP group.

**Figure 3:**
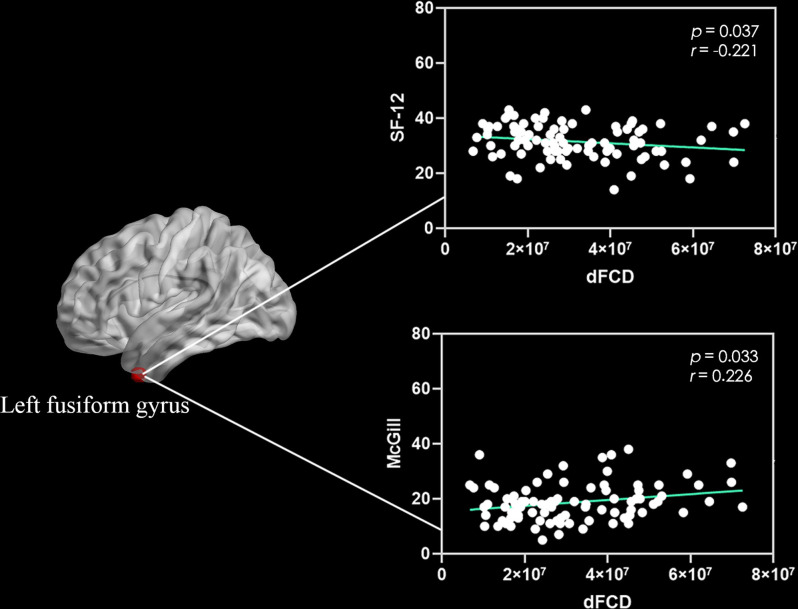
Correlation between dynamic functional connectivity density (dFCD) variability in the left fusiform gyrus with McGill scores and SF-12 scores in the CNP group. SF-12, short form 12 questionnaire.

**Figure 4:**
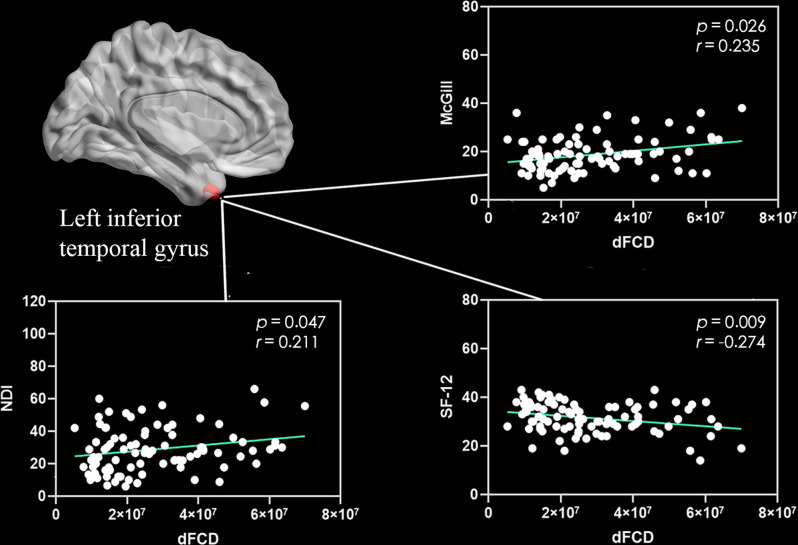
Correlation between dynamic functional connectivity density (dFCD) variability in the left inferior temporal gyrus and right inferior temporal gyrus with McGill scores, neck disability index (NDI) scores, and SF-12 scores of the CNP Group. NDI, neck disability index; SF-12, short form 12 questionnaire.

**Figure 5:**
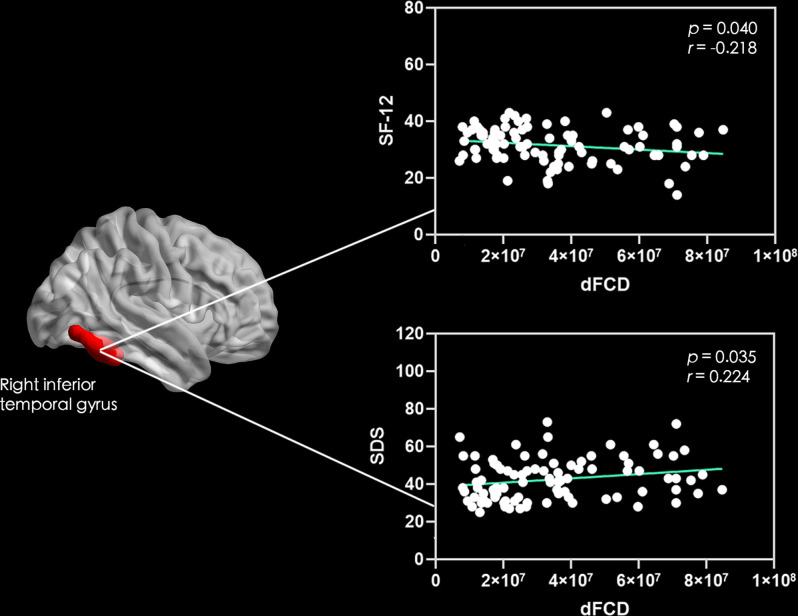
Correlation between dynamic functional connectivity density (dFCD) variability in the right inferior temporal gyrus with SF-12 scores and self-rating depression scale (SDS) scores of the CNP Group. SF-12, short form 12 questionnaire; SDS, self-rating depression scale.

Correlation analyses showed that the excessive dFCD variability of the left medial and paracingulate gyrus (Figure 2A) and the right anterior cingulate and paracingulate gyrus (Figure 2B) were negatively correlated with the CNP’s duration. The dFCD variation in the CNP patients’ left fusiform gyrus was positively correlated with McGill scores and negatively correlated with SF-12 scores (Figure 3). The dFCD variation in the left inferior temporal gyrus was positively correlated with McGill scores, NDI scores, and SF-12 scores of CNP patients (Figure 4). The dFCD variation in the right inferior temporal gyrus was positively correlated with the SDS scores and SF-12 scores of CNP patients (Figure 5). No significant correlations were observed between the dFCD variability in other ROIs and VAS scores or SAS scores in CNP groups.

## Discussion

We investigated the dFCD of CNP patients and HCs and further explored the potential associations of abnormal dFCD variability with CNP patients’ pain characteristics since dFCD can reflect the ability of brain areas or voxels to integrate information. Our study found that CNP patients had increased dFCD values compared with those of HCs, mainly in the ACC, occipital lobe, temporal lobe, and cerebellum. Our results suggested that the anterior cingulate and paracingulate gyrus, left fusiform gyrus, and inferior temporal gyrus was significantly correlated with the characteristics (such as the degree of neck pain and impact of neck pain on emotion and daily life) of CNP patients and implied that these areas are important to CNP.

The cingulate gyrus has an important role in the limbic system because it is not only the core brain area of the default network but also the main component of the medial pain system. Its anterior part, the anterior cingulate gyrus, is mainly responsible for pain processing. The ACC has the core position in the integration center responsible for pain coding (Budell et al., 2015). The ACC is involved in pain formation, pain coding, and avoidance responses to pain behaviors. With nociceptive stimuli, the anterior cingulate gyrus integrates pain sensations, headache cognition, painful emotions, and other information to produce multisystem-specific responses to pain stimuli (including emotions, attention, movement, and autonomic nervous activity). When patients with chronic pain were compared with HCs (Apkarian et al., 2004), the ACC contained a high concentration of opioid receptor binding sites, thus proving that the ACC has a key role in pain formation and management. Furthermore, the increase in functional activation is not limited to the primary sensorimotor cortex; it also occurs in the supplementary motor area, anterior cingulate, thalamus, basal ganglia, and cerebellum (Hrabálek et al., 2014). These areas are ostensibly recruited to assist the primary sensorimotor cortex to complete motor tasks. This was reflected in the enhancement of dFCD between these regions during this study. In terms of static functional connectivity, previous results have suggested increased functional connectivity within sensorimotor regions with increasing neurological impairment and decreased connectivity among the cerebellum, putamen, thalamus, anterior and posterior cingulate, and frontal lobe regions in cervical spondylosis patients (Woodworth et al., 2018). During this study, CNP patients exhibited increased dFCD variability in the cluster of the cerebellum, ACC, occipital lobe, and temporal lobe compared with HCs. Previous studies have shown that areas involved in high-level functions usually show higher dFC, which may be related to the flexible coupling of regulatory systems with other brain networks (Gonzalez-Castillo et al., 2014). This seems to explain the increased dFCD variability in the relevant brain regions shown by our results.

The temporal lobe plays an important role in human mental activities and perceptual functions (Behrmann et al., 2016) are associated with high-level mental activities such as memory and emotion, which have received attention from those studying brain function with chronic pain. One study (Kakigi et al., 2004) reported that the temporal lobe is mainly responsible for pain cognition and emotional response. The middle temporal gyrus (MTG), located in the middle of the temporal lobe between the superior temporal sulcus and inferior temporal sulcus, is the center of language and auditory processing and is also involved in memory, learning, and emotion (Caetano et al., 2007). Studies have found that the dysfunction of the MTG is associated with cluster headache, migraine, chronic low back pain, chronic shoulder pain, and other chronic pain (Absinta et al., 2012). Repeated pain leads to decreased neural activity in brain regions, resulting in negative emotions (Qi et al., 2016). Therefore, the increased dFCD value of the left superior temporal gyrus in CNP with the characteristics of recurrent pain episodes may be related to pain accumulation and negative emotions caused by chronic pain.

Changes in the limbic system’s function and structure may be associated with chronic pain. One study (Mao et al., 2016) found that patients with somatoform pain disorders have abnormal activation in the right cerebellum. During the early stages of this study, it was found that CNP patients’ SDS scores were generally higher than normal, indicating that these patients developed a negative mood (depression). The cerebellum has an important role in the initial discrimination of pain perception, emotions, and information. This may be because the cerebellum is related to nociceptive perception and emotional experiences caused by neck pain. Nociceptive perception and emotional experiences caused by pain are related; with noxious and painful stimuli, the cerebellum responds through emotion, cognitive control, and motor control (Moulton et al., 2010). Studies have suggested that the cerebellum is involved in preparing for defense against nociceptive stimuli and has important roles in the perception and transmission of nociceptive information and control of nociceptive movement; furthermore, it is of major importance to the positioning of the nociceptive stimuli (Helmchen et al., 2004). Therefore, we speculate that the increased dFCD value of the right cerebellum may be associated with the pain location and lead to negative emotions in CNP patients.

The occipital cortex integrates the somatosensory system, vision, and hearing. It comprises an important visual processing center of the brain, especially the middle occipital gyrus. It accepts and participates in basic visual processing and can be regulated by factors such as attention (Xu et al., 2014). Damage to the occipital lobe causes visual problems, memory impairment, and motor perception impairment. One study found that chronic pain caused a decrease in the gray matter volume of the occipital cortex. Our previous studies of migraine (Zhang et al., 2021) found that the ReHo value of the left middle occipital gyrus or right middle occipital gyrus decreased after acupuncture treatment and that it may be related to the relief of photophobia and the reduction of pain-associated symptoms in migraine patients. These results suggest the occipital lobe’s importance to the field of pain and help explain the occipital lobe’s higher dFCD variability in CNP.

The correlation analysis indicated that the longer the course of the disease, the lower the dFCD value of the right anterior cingulate and paracingulate gyrus and left medial and paracingulate gyrus, suggesting that connectivity of these brain regions gradually decreases with disease progression. The ACC has important roles in pain formation, pain coding, and the avoidance response to pain behavior. Furthermore, it can create a multisystem-specific response to pain when it occurs (Budell et al., 2015). When the anterior cingulate gyrus is stimulated by injury, it will produce inattention, apathy, emotional abnormalities, and autonomic dysfunction. Patients with chronic headaches caused by whiplash had a decreased volume of the anterior cingulate gyrus and dorsolateral prefrontal gray matter 3 months after injury (Obermann et al., 2009). We speculate that the abnormal dFCD of the anterior cingulate gyrus may be an important central feature of CNP. The dFCD values in these regions were negatively correlated with the disease course, which may indicate that, with the accumulation of painful stimulation, the dFCD in these brain regions decreases more severely and the key role of the ACC decreases. This may be related to the pain tolerance caused by CNP.

For CNP patients, the McGill score was positively correlated with dFCD variability in the left fusiform gyrus and left inferior temporal gyrus. Similarly, the NDI score was positively correlated with excessive dFCD variability in the left inferior temporal gyrus, and the SDS score was positively correlated with the excessive variability of dFCD in the right inferior temporal gyrus; however, the SF-12 score was negatively correlated with dFCD variability in the left fusiform gyrus and inferior temporal gyrus. The McGill score assesses the degree of pain experienced by patients, the NDI assesses the degree of cervical spine dysfunction, and the SDS assesses depression. The higher the scores of these three assessments, the more severe the illness. The SF-12 scale evaluates the quality of life of patients; the higher the score, the better the quality of life. With increased degrees of pain, neck dysfunction, and negative emotions, the dFCD of the right inferior temporal gyrus/fusiform gyrus may increase significantly, indicating that dFCD in the aforementioned brain regions can objectively reflect the degrees of pain, neck dysfunction, and negative emotions. Previous studies have found a significant increase in spontaneous nerve activity in the bilateral temporal lobes, fusiform gyrus, and left parahippocampal gyrus of patients with lumbar intervertebral disc protrusion (Zhang et al., 2017). The temporal lobe has an important effect on memory and emotions. Therefore, the memory and emotional regulation of pain patients are abnormal, which may increase the temporal lobe dFCD. Baliki et al. (2006) and Zhang et al. (2017) showed a correlation between the neural activity of the pain-related brain area and the degree of pain, and that the adjustment of pain was not independent; instead, it involved the coordination of multiple brain areas.

The age difference between HCs and CNP patients was statistically significant (*p* < 0.05), possibly because the incidence of neck pain occurred in younger individuals as a result of their occupation and lifestyle (Alvin et al., 2014).

The latest study on the gender gap in chronic pain found that significant gender pain inequalities exist across Europe whereby women (62.3%) experience more pain than men (55.5%). These inequalities were greatest for back/neck pain (Bimpong et al., 2022). The severity, frequency, range, and duration of pain were higher in women than in men over the same disease (Affaitati et al., 2011). Persistent chronic pain is bound to lead to psychological and mental changes, accompanied by negative emotions, especially in women (Wiech and Tracey, 2009). There are many factors contributing to this difference, and estrogen may play a key role (Hurley and Adams, 2008).

According to the results of our study on the dynamic functional alterations in the brain regions of CNP patients, the dFCD in these brain areas can objectively reflect the degree of pain and negative emotions caused by pain to a certain extent, which has a certain guiding significance for our clinical diagnosis of CNP. Moreover, site-specific treatment can be carried out or the mechanism of therapeutic effect can be detected according to these specific brain regions.

There were several limitations to this study. Although dFCD has been widely used in previous clinical studies, such as studies of psychiatric disorders, its expression with pain has not been sufficiently understood. The use of analgesics by some patients may confuse resting-state imaging and clinical behavioral test results. Although we evaluated dFCD in drug-naive patients during a relatively limited time, these findings need to be repeated in a larger, homogeneous, prospective study. Finally, despite short-distance and long-distance connections, dFCD is calculated as the average of all connections in the entire brain. In future studies, FCD with short-range and long-range connections should be calculated separately.

In conclusion, the results of the combination of voxel-based FCD mapping and sliding windows correlation analysis demonstrated abnormal functional dynamics in CNP. The dFCD values of the ACC, occipital lobe, temporal lobe, and cerebellum had relatively high temporal variance, which might be related to important aspects of the pain network and emotional network. Among them, the dFCD in the brain region that is related to pain processing and emotional feedback can reflect the degrees of pain, neck dysfunction, and negative emotions. Our results supported the dynamic functional alterations in the brain regions of CNP patients, and the dFCD of these brain regions could become neuroimaging markers for objectively reflecting pain to a certain extent. Chronic pain may cause changes in pain processing and emotional feedback, thus highlighting the link between dynamic neural communication in brain regions and disease conditions, deepening our understanding of chronic pain diseases, and guiding clinical practice.

## Data Availability Statement

The raw data supporting the conclusions of this article will be made available by the authors, without undue reservation.

## Ethics Statement

The studies involving human participants were reviewed and approved by Sichuan Regional Ethics Review Committee on Traditional Chinese Medicine. The patients/participants provided their written informed consent to participate in this study.

## Author Contributions

Author contributions included conception and study design (XN, JZ, and LZ), data collection or acquisition (MS, LW, QZ, and ZW), statistical analysis (TX and XW), interpretation of results (HL, YH, TX, and LZ), drafting the manuscript work or revising it critically for important intellectual content (XN, JZ, and LZ), and approval of final version to be published and agreement to be accountable for the integrity and accuracy of all aspects of the work (All authors). All authors contributed to the article and approved the submitted version.

## Conflict of Interest

The authors declare that the research was conducted in the absence of any commercial or financial relationships that could be construed as a potential conflict of interest.

## Publisher’s Note

All claims expressed in this article are solely those of the authors and do not necessarily represent those of their affiliated organizations, or those of the publisher, the editors and the reviewers. Any product that may be evaluated in this article, or claim that may be made by its manufacturer, is not guaranteed or endorsed by the publisher.

## Footnotes

^1^ http://www.nitrc.org/projects/mricron

^2^ http://rfmri.org/dpabi

^3^ http://www.chictr.org.cn/index.aspx
